# Supplementary material for: Costs and health benefits of the rural energy transition to carbon neutrality in China
Source: Nat Commun. 2023 Sep 29;14:6101. doi: 10.1038/s41467-023-41707-7 (PMC10541415; doi:10.1038/s41467-023-41707-7)
Supplement: Supplementary file 1 — Supplementary Information [file 41467_2023_41707_MOESM1_ESM.pdf]

Costs and health benefits of the rural energy transition to carbon neutrality in China

Teng Ma[1], Silu Zhang[1], Yilong Xiao[1], Xiaorui Liu[1], Minghao Wang[2], Kai Wu[1], Guofeng Shen[3,4], Chen Huang[1,5], Yan Ru Fang[1]* & Yang Xie[2,6]*

[1] College of Environmental Sciences and Engineering, Peking University, 100871 Beijing, China

[2] School of Economics and Management, Beihang University, 100191 Beijing, China

[3] College of Urban and Environmental Sciences, Laboratory for Earth Surface Processes, Peking University, 100871 Beijing, China.

[4] Institute of Carbon Neutrality, Peking University, Beijing, 100871, China

[5] International Institute for Applied Systems Analysis (IIASA), Laxenburg, Austria

[6] Laboratory for Low-carbon Intelligent Governance, Beihang University, 100191 Beijing, China

*Corresponding to

Dr. Yan Ru Fang, College of Environmental Sciences and Engineering, Peking University, 100871 Beijing; China, fyr000@126.com

Dr. Yang Xie, School of Economics and Management, Beihang University, 100191 Beijing, China; xieyangdaisy@buaa.edu.cn

Content

Part A. Supplementary methods......3

A.1 The residential module of IMED|TEC model......3

A.2 GAINS model......7

A.3 IMED|HEL model......8

Part B. Supplementary results......12

Part C. Sensitivity analysis......16

C.1 Methods......16

C.2 Results......18

Supplementary reference......24

Part A. Supplementary methods

A.1 The residential module of IMED|TEC model

IMED|TEC model is developed by the Laboratory of Energy & Environmental Economics and Policy (LEEEP) at Peking University, which focuses on technology selection with the least system cost, consisting of technology investment, operation and maintenance cost, and energy cost. The residential module of the IMED|TEC model is a multi-province bottom-up energy system optimization model in which 31 provincial administrative regions in China (Hong Kong, Macau and Taiwan regions are not included due to lack of data) are considered and each has its own technology-selection module. By taking 2014 as the base year and one year as a cycle, the residential module of the IMED|TEC model allows for simulating the bottom-up technology-selection process at the provincial level and exploring cost-effective decarbonization pathways under given assumptions and constraints to meet the exogenous service demand, such as rural cooking and heating service demand. Detailed information about rural cooking and heating of IMED|TEC is shown in Supplementary Table 1.

Supplementary Table 1. Technologies for rural cooking and heating in IMED|TEC and parameters

Number	Technology	Energy	Service	Efficiency
1	Straw cooking range	Straw	Cooking	0.21
2	Firewood cooking range	Firewood	Cooking	0.24
3	Coal cooking range	Coal	Cooking	0.28
4	Biogas cooking range	Biogas	Cooking	0.45
5	LPG cooking range	LPG	Cooking	0.55
6	Electric cooking range	Electricity	Cooking	0.85
7	Straw stove	Straw	Heating	0.24
8	Firewood stove	Firewood	Heating	0.28
9	Coal stove	Coal	Heating	0.39
10	Improved coal stove	Clean coal	Heating	0.71
11	Biogas stove	Biogas	Heating	0.57
12	Natural gas stove	Natural gas	Heating	0.85
13	Resistance heater	Electricity	Heating	0.90
14	Air-to-air heat pump	Electricity	Heating	0.07×ambient temperature (°C) +2.69

A.1.1 Rural cooking and heating service demand estimation

For certain regions, rural cooking or heating service demand can be calculated by multiplying the future rural people under shared socioeconomic pathways (SSP) and future service demand per capita annually. Therefore, projected rural people reduction reduces cooking and heating demand, leading to lower rural energy consumption and CO_2 emissions even with a fixed energy mix. Future

service demand per capita is estimated based on a statistical relationship between historical service demand per capita and GDP per capita, where historical service demand per capita is estimated by dividing historical residential energy consumption (2005-2014) by device efficiency. Specifically, it is found that during 2005-2014, the cooking demand per capita in almost all regions and heating demand per capita in the southern regions did not notably change. In contrast, the heating demand per capita in most northern regions was positively correlated with GDP per capita. Therefore, we assume that the future cooking demand per capita in all regions and heating demand per capita in the southern regions remain at the 2014 level, whereas the future heating demand per capita in certain northern regions is adjusted according to per capita GDP. Notably, the projected provincial service demand considers regional socioeconomic and climatic heterogeneity based on the above sub-national estimation approach.

Data. The primary data about historical rural residential energy use for building the provincial rural cooking and heating module of the IMED|TEC model is based on a representative nationwide residential energy survey conducted by Tao et al.[1, 2]. The parameters for household technology efficiency come from several previous studies[3, 4, 5, 6, 7, 8](Supplementary Table 1). Especially, we considered the regionally adjusted efficiency of the air-to-air heat pump in the modeling[3, 9]. The predicted data of China's people and GDP during 2014-2060 under SSP2 comes from Chen et al.[10] and Jiang et al.[11], respectively.

A.1.2 Technology selection part

Taking meeting the end-use service demand as the premise, the objective function for technology selection in IMED|TEC is to minimize the total energy system cost. IMED|TEC is formulated as a linear program, considering service demand constraints, CO_2 and air pollutants emission constraints, technology share ratio constraints, energy use constraints, and dynamic balance of technology stock quantity under given assumptions.

(1) Service demand constraints

In the rural residential sector, the total amount of service provided by technologies in operation should meet cooking and heating demand, as shown in Eq. 1.

$$\sum_{t} X_t \cdot A_{sd,t} \geq SVD_{sd} \quad (1)$$

Where, SVD_{sd} represents the service demand; X_t represents the operation quantity of technology t; $A_{sd,t}$ represents the service demand sd provided by per unit of technology t, i.e., technology efficiency (Supplementary Table 1-2). Especially, the efficiency of AAHP is provincially adjusted based on ambient temperatures.

Supplementary Table 2. Provincial efficiency of AAHP and ambient temperatures

Provinces/ municipalities/ autonomous regions	Average ambient temperatures during the heating season*	Efficiency of AAHP

Anhui	6.41	3.14
Beijing	0.4	2.72
Chongqing	10	3.39
Fujian	13.94	3.67
Gansu	-0.61	2.65
Guangdong	16.12	3.82
Guangxi	14.98	3.74
Guizhou	6.93	3.18
Hainan	20	4.09
Hebei	1.9	2.82
Heilongjiang	-12.33	1.83
Henan	4.37	3.00
Hubei	7.22	3.2
Hunan	8.55	3.29
Inner Mongolia	-6.47	2.24
Jiangsu	6.48	3.14
Jiangxi	9.18	3.33
Jilin	-9.86	2.00
Liaoning	-6.83	2.21
Ningxia	-2.84	2.49
Qinghai	-4.79	2.35
Shaanxi	3.51	2.94
Shandong	3.01	2.9
Shanghai	8.25	3.27
Shanxi	-1.23	2.6
Sichuan	8.78	3.3
Tianjin	0.41	2.72
Tibet	2.08	2.84
Xinjiang	-7.83	2.14
Yunnan	10.88	3.45
Zhejiang	8.22	3.27

*Average ambient temperatures during the heating seasons from 2010-2017 (November, 2010-Feburary, 2018) come from the national meteorological observatory database. This database is managed by China Meteorological Data Service Center.

(2) Technology share ratio constraints to certain services

The technology penetration for certain service sd refers to the maximum proportion $\theta_{t,sd}^{max}$ and minimum proportion $\theta_{t,sd}^{min}$ of each technology in all technologies T_{sd}, as shown in Eq. 2.

$$\theta_{t,sd}^{min} \cdot \sum_{t' \in T_{sd}} X_t \cdot A_{sd,t} \leq X_t \cdot A_{sd,t} \leq \theta_{t,sd}^{max} \cdot \sum_{t' \in T_{sd}} X_t \cdot A_{sd,t} \quad (2)$$

Where, $\theta_{t,sd}^{max}$ and $\theta_{t,sd}^{min}$ represent the maximum and minimum proportion of technology t in certain service sd, respectively; T_{sd} represents the technology groups for service sd.

(3) CO_2 and air pollutants emission constraints

The emissions of CO_2 or air pollutants g from all technologies must not exceed its maximum emission constraint, as shown in Eq. 3.

$$\sum_t X_t \cdot EM_{t,g} \leq \widehat{Q_g} \quad (3)$$

Where, $EM_{t,g}$ represents the emissions of gas g from per unit of technology t in operation; $\widehat{Q_g}$ represents the maximum emission constraint of gas g.

(4) Energy use constraints

Consider the availability of energy, the total amount of energy use E_e refers to the maximum and minimum constraints.

$$E_e^{min} \leq E_e \leq E_e^{max} (4)$$

Where, E_e^{min} and E_e^{max} represent the maximum and minimum supply of energy e, respectively, as shown in Eq. 4.

(5) Dynamic balance of technology stock quantity

The stock quantity of technology t is calculated by Eq. 5.

$$S_t = SS_t \cdot \left(1 - \frac{1}{T_t}\right) - w_t + r_t \quad (5)$$

Where, S_t repreents the operating quantity of technology t in the current year and SS_t represents the remaining stock quantity technology t in the previous year, respectively; w_t represents the retired stock quantity of technology t before lifetime; r_t represents recruited quantity of technology t to meet the service demand. S_t and SS_t follow the Weibull distribution. $SS_t\left(1 - \frac{1}{T_t}\right)$ represents the remaining stock quantity from the previous year to the current year after natural depreciation.

Data. The parameters for household device cost come from several previous studies[3, 6, 7, 8, 12]. Notably, we set the costs of contemporary technologies (AAHP, electrical cooking range, LPG cooking range and natural gas stove) to decline linearly by 20% between 2020 and 2060[13]. Energy price, CO_2 and air pollutants emission factors are shown in Supplementary Table 3 - 4.

Based on the summary of household fuels price by Zhou et al.[3] and predicted relative energy prices under SSP2[14, 15], we consider the change of household commercial energy prices in the future (Supplementary Table 3). Carbon emission limits lead to higher fossil energy price and lower electricity price, which is consistent with the current trend of technological development. As firewood, straw and biogas are free to collect and thus have no energy price, we don't list them in Supplementary Table 3.

Supplementary Table 3. Commercial energy prices without subsidies for the residential sector during 2014-2060 (US$/toe, 2020 price level)

Fuel	2014	2020	2030	2040	2050	2060
coal	140	146	171	206	257	320
clean coal	280	291	342	412	512	638

NG/ LPG	370	385	429	474	540	611
electricity	770	755	701	639	570	508

Supplementary Table 4. Emission factors

Fuel	CO_2 (kg/kgoe)	SO_2 (g/kgoe)	NO_x (g/kgoe)	$PM_{2.5}$ (g/kgoe)
Coal	3.96	24.16	3.22	16.10
Clean coal	3.47	3.38	1.90	0.14
Firewood	0.00	0.25	3.50	19.25
Straw	0.00	0.83	5.54	18.56
Biogas	0.14	1.68	0.48	0.24
Natural gas	2.35	0.42	0.84	0.00
LPG	2.64	0.17	1.75	0.42

Data source: Zhou et al.[3], Xing et al.[6], Meng et al.[16] and Wang et al.[17]

A.2 GAINS model

The input data is different energy consumption data at the provincial level from the IMED|TEC model. The sectoral mapping between IMED|TEC model and the GAINS model is shown in Supplementary Table 5. The scenarios selected by other sectors were the WEO2020 scenario built into GAINS.

Supplementary Table 5. The sectoral mapping between the IMED|TEC model and GAINS model

IMED|TEC	GAINS
Coal	HC2
Clean coal	HC3
Straw	ARD
Firewood	FWD
Electricity	ELE
Liquefied petroleum gas	LPG
Natural gas	GAS
Biogas	BIOG

GAINS model allows estimation of air pollutant emissions by using Eq. 6.

$$E_{i,p} = \sum_k \sum_m A_{i,k} \times EF_{i,k,p} \times (1 - \eta_{i,m}) \times x_{i,k,m,p} \quad (6)$$

Emissions-generating economic activities are organized into activity pathways. GAINS divides activity data into five groups: Energy (ENE), Mobile sources (MOB), Agriculture (AGR), Process (PROC), and VOC-specific (VOC).

Where *i, k, m, p* represent region, activity type, abatement measure and pollutant, respectively; $E_{i,p}$ denotes emissions of pollutant *p* in region *i*; $A_{i,k}$ denotes the activity of type *k* (e.g., coal consumption in the rural residential sector) in region *i*; $EF_{i,k,m,p}$ denotes emission factor of pollutant *p* for activity *k* in country *i* without the application of control measures; $\eta_{i,m}$ denotes removal efficiency of control measure *m* in region *i*; $x_{i,k,m,p}$ denotes the technology penetration rate, which means the share of the total activity of type *k* in country *i* to which a control measure *m* for pollutant

p is applied. As for the emission factors of different air pollutants, there are different equations.

The calculation of $PM_{2.5}$ concentration is based on a simplified source-sink matrix. The atmospheric transport coefficient is statistically obtained based on perturbation simulation of the global EMEP chemical transport model[18]. The relationship between emissions and concentration is linear, and the error is usually less than 1μg/m^3 (Eq. 7).

$$C(P_{2.5})_i = \sum[\pi_i \times \text{Em }(PPM) + \sigma_i \times \text{Em }(\text{SO}_2) + \alpha_i \times \text{Em }(\text{NO}_X) + \beta_i \times \text{Em }(NH_3) + \gamma_i \times \text{Em }(\text{VOC}_i)] + \mu_i \quad (7)$$

Where C($PM_{2.5}$)$_i$ is the $PM_{2.5}$ concentration in grid cell i; Em(PPM) represents the total primary $PM_{2.5}$. The constants π, σ, a, β, γ are the source-receptor matrices for the corresponding pollutant's contribution to the $PM_{2.5}$ concentration and the constants μ_i are grid cell-specific.

A.3 IMED|HEL model

Using the health model to quantify air pollution-related mortality, morbidity, work time loss, health expenditures, and VSL. The health impact assessment model integrated several exposure-response functions (ERFs) from global epidemiological studies, including the Log-Linear equation, non-linear IER function and GEMM function. However, the latest Global Exposure Mortality Model (GEMM) was used in this study.

GEMM was inspired by the Log-Linear (LL) model, a commonly used method to estimate excess deaths from exposure to ambient $PM_{2.5}$. The association between concentrations of $PM_{2.5}$ and mortality for the GEMM analysis of a specific cohort is described by a class of hazard ratio functions in Eq. 8:

$$RR_{i,y,k,a} = \exp\{\theta_{k,a} \log\left(\frac{z_{iy}}{\alpha_{k,a}} + 1\right) \left[\frac{1}{1 + \exp\left(-\frac{z_{iy} - \mu_{k,a}}{v_{k,a}}\right)}\right] \quad (8)$$

Where, $z_{ij} = C_{ij} - cf$, C_{ij} is the ambient concentration of $PM_{2.5}$ in region i and year y, and cf is the counterfactual concentration of $PM_{2.5}$ below which there is assumed to be no additional risk. In the GEMM, θ and its SE control the slope of a non-linear regression that represents the relationship between exposure concentration and RR, and α defines the curvature of the model. Burnett et al. (2018) reported age-specific parameters of θ, α, μ, v from 41 cohort studies from 16 countries[19]. The theoretical counterfactual minimum risk exposure $PM_{2.5}$ concentration reported in the recent GBD 2018 study of 2.4 μg/m^3 is used in the current study[19].

The number of health endpoints is estimated by multiplying RR with the people and reported cause-specific mortality rate (Eq. 9).

$$EP_{i,y,k} = \sum_a \frac{RR(C)_{i,y,k,a} - 1}{RR(C)_{i,y,k,a}} \times I_{i,y,a} \times P_{i,y,k} \quad (9)$$

where $P_{i,y,k}$ is the people number of region i in a certain year y and scenario k. $I_{i,y,a}$ is the baseline mortality rate of disease a in year y in region i. The baseline mortality rate used was derived from the previous study[20].

We use the elasticity coefficient to calculate the different value of statistical life (VSL) calibration values corresponding to different GDP per capita levels of China's provinces in 2035 and 2050, as shown in Eq. 10 and Supplementary Table 6.

$$VSL_{r,year,type} = VSL_{China,2015,type} \times \left(\frac{GDPper_{r,year}}{GDPper_{China,2015}}\right)^{elasticity} \quad (10)$$

Where, $VSL_{r,year,type}$ is the calibration medium/high/low VSL of China's provinces in 2035 and 2050, $VSL_{China,2015,type}$ is the medium/high/low VSL of China in 2015, estimated by Jin et al.[21], $GDPper_{China,2015}$ and $GDPper_{r,2050}$ are the per capita GDP of China in 2015 and the per capita GDP of China's provinces in 2050 under SSP2[11], respectively. $elasticity$ is the elasticity coefficient of VSL on per capita GDP, and we adopt the reference value of 0.8[22].

Supplementary Table 6. Provincial VSL in 2035 and 2050 at the medium/high/low level (million US$, 2020 price level)

Region	2035			2050		
	High VSL	Medium VSL	Low VSL	High VSL	Medium VSL	Low VSL
Anhui	1.59	0.95	0.70	2.23	1.34	0.98
Beijing	3.41	2.05	1.50	4.30	2.58	1.89
Chongqing	2.21	1.33	0.97	2.71	1.63	1.19
Fujian	3.38	2.03	1.49	4.79	2.88	2.11
Gansu	1.30	0.78	0.57	1.96	1.18	0.86
Guangdong	3.15	1.89	1.39	3.93	2.36	1.73
Guangxi	1.13	0.68	0.50	1.28	0.77	0.56
Guizhou	0.96	0.57	0.42	1.02	0.61	0.45
Hainan	1.92	1.15	0.84	2.34	1.40	1.03
Hebei	2.33	1.40	1.03	3.03	1.82	1.33
Heilongjiang	1.59	0.96	0.70	2.08	1.25	0.92
Henan	2.50	1.50	1.10	3.50	2.10	1.54
Hubei	2.39	1.43	1.05	3.17	1.90	1.40
Hunan	1.83	1.10	0.80	2.54	1.52	1.12
Inner Mongolia	3.13	1.88	1.38	3.67	2.20	1.62
Jiangsu	4.82	2.89	2.12	5.68	3.41	2.50
Jiangxi	1.72	1.03	0.76	2.05	1.23	0.90
Jilin	2.12	1.27	0.93	3.16	1.90	1.39
Liaoning	2.75	1.65	1.21	4.05	2.43	1.78
Ningxia	1.70	1.02	0.75	2.31	1.39	1.02
Qinghai	1.61	0.96	0.71	2.13	1.28	0.94
Shaanxi	1.77	1.06	0.78	2.71	1.62	1.19
Shandong	4.17	2.50	1.83	5.95	3.57	2.62
Shanghai	7.07	4.24	3.11	9.94	5.96	4.37
Shanxi	1.43	0.86	0.63	1.94	1.16	0.85
Sichuan	2.19	1.32	0.96	2.68	1.61	1.18
Tianjin	4.03	2.42	1.77	5.40	3.24	2.38
Xinjiang	1.40	0.84	0.62	1.84	1.10	0.81
Tibet	1.25	0.75	0.55	1.49	0.90	0.66
Yunnan	1.40	0.84	0.62	1.61	0.97	0.71

Zhejiang	4.72	2.83	2.08	6.50	3.90	2.86

Supplementary Table 7. Comparison of the estimated air pollutants emissions and energy consumption of rural cooking and heating in 2014 of this study and from the other literature.

Item	This study	Tao et al. [1 a]	Yun et al.[23]	Wu et al. [5, 24 b]
SO_2 (Mton)	1.21	1.20	1.3	-
NO_x (Mton)	0.58	0.64	-	-
$PM_{2.5}$ (Mton)	2.56	2.72	2.4	-
Energy use (Mtoe)	151	184	148	-
Energy use per capita (kgoe)	246	-	-	236
Share of biomass use (%)	63	67	64	61

[a,] the data is for the year 2012. Energy use data is estimated by aggregating various fuel use. It is important to note that rural residential energy consumption for cooking and heating, as estimated by Tao et al., experienced a decrease of 60 Mtoe between 2007 and 2012.
[b,] the data is for the year 2013. Energy use per capita is estimated by considering the total rural household energy consumption per capita (267 kgoe) and the proportion of energy use dedicated to cooking and heating (88%). The data regarding the share of biomass use incorporates biogas, which refers to the overall energy consumption rather than solely the energy consumption for cooking and heating.

Considering provincial socioeconomic disparity, we divided the 31 provinces into two categories, the former with a higher GDP per capita than the national average (including 10 provinces of Beijing, Fujian, Guangdong, Inner Mongolia, Jiangsu, Liaoning, Shandong, Shanghai, Tianjin and Zhejiang), and the latter with a lower GDP per capita than the national average in 2020[10, 11]. For the former, the direct CO_2 emissions cap of rural cooking and heating (RCH) would reduce by 35% and 95% in 2035 and 2060, respectively, compared with 2015 at the provincial level, which follows the 1.5°C target of China's residential sector[25]. For the latter, the direct CO_2 emissions cap of RCH would reduce by 25% and 90% in 2035 and 2060, respectively, compared with 2015 at the provincial level. The detailed provincial CO_2 cap of RCH during 2020-2060 is shown in Table A.8.

Supplementary Table 8. Provincial CO_2 cap of rural cooking and heating during 2020-2060 (Mton)

Region	Abbreviation	2020	2035	2060
Anhui	AH	4.905	2.391	0.242
Beijing	BJ	1.043	0.298	0.005
Chongqing	CQ	0.772	0.330	0.030
Fujian	FJ	1.246	0.498	0.025
Gansu	GS	14.255	6.937	0.610
Guangdong	GD	1.525	0.714	0.040
Guangxi	GX	0.781	0.405	0.044
Guizhou	GZ	8.215	4.780	0.508
Hainan	HN	0.076	0.042	0.005
Hebei	HeB	23.120	10.597	1.107

Heilongjiang	HLJ	8.813	4.947	0.407
Henan	HeN	11.420	5.528	0.566
Hubei	HuB	4.332	2.302	0.223
Hunan	HuN	7.956	3.967	0.397
Inner Mongolia	NM	12.689	4.939	0.224
Jilin	JS	7.978	4.174	0.350
Jiangsu	JX	3.802	1.651	0.093
Jiangxi	JL	2.325	1.174	0.122
Liaoning	LN	7.361	3.013	0.132
Ningxia	NX	2.437	1.395	0.156
Qinghai	QH	2.700	1.492	0.139
Shaanxi	SaX	9.712	4.600	0.421
Shandong	SD	22.991	9.648	0.517
Shanghai	SH	0.183	0.069	0.002
Shanxi	SX	31.102	18.528	1.786
Sichuan	SC	6.653	3.245	0.275
Tibet	TJ	0.178	0.093	0.009
Tianjin	XZ	1.119	0.404	0.011
Xinjiang	XJ	13.838	9.044	1.096
Yunnan	YN	2.947	1.582	0.148
Zhejiang	ZJ	2.452	1.079	0.052

Taking the "Qin Mountain-Huai River" as the boundary, we consider the provinces to the north as the northern region and the provinces to the south as the southern region.

Supplementary Table 9. Northern and southern provinces

Region	Provinces
South	Anhui, Chongqing, Fujian, Guangdong, Guangxi, Guizhou, Hainan, Hubei, Hunan, Jiangsu, Jiangxi, Shanghai, Sichuan, Tibet, Yunnan, Zhejiang
North	Beijing, Gansu, Hebei, Heilongjiang, Henan, Inner Mongolia, Jilin, Liaoning, Ningxia, Qinghai, Shaanxi, Shandong, Shanxi, Tianjin, Xinjiang

Part B. Supplementary results

Supplementary Fig 1

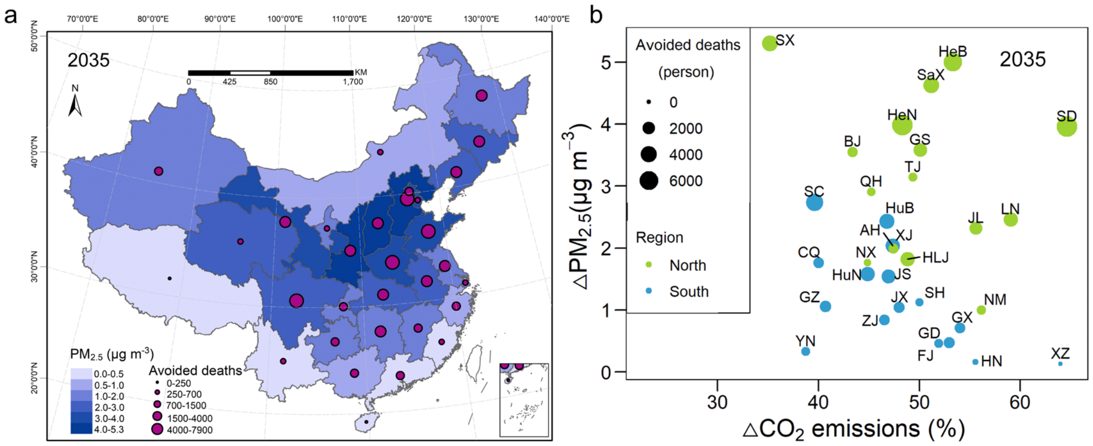

Supplementary Fig 1. (a) Reduction in ambient $PM_{2.5}$ concentrations and avoided premature deaths in carbon neutrality scenario (CNS) relative to baseline scenario (BaU) in 2035.; (b) regional disparity in carbon reduction, air quality improvement, and avoided $PM_{2.5}$-associated premature deaths in 2035. In (a), blue shades represent reductions in ambient $PM_{2.5}$ concentrations, and red dots indicate avoided $PM_{2.5}$-associated premature deaths in CNS relative to BaU. In (b), blue and dark green dots represent southern and northern provincial regions, respectively, with dot size reflecting provincial avoided $PM_{2.5}$-associated premature deaths. Base map data adapted from GS(2020)4619, http://bzdt.ch.mnr.gov.cn/.

Supplementary Fig 2

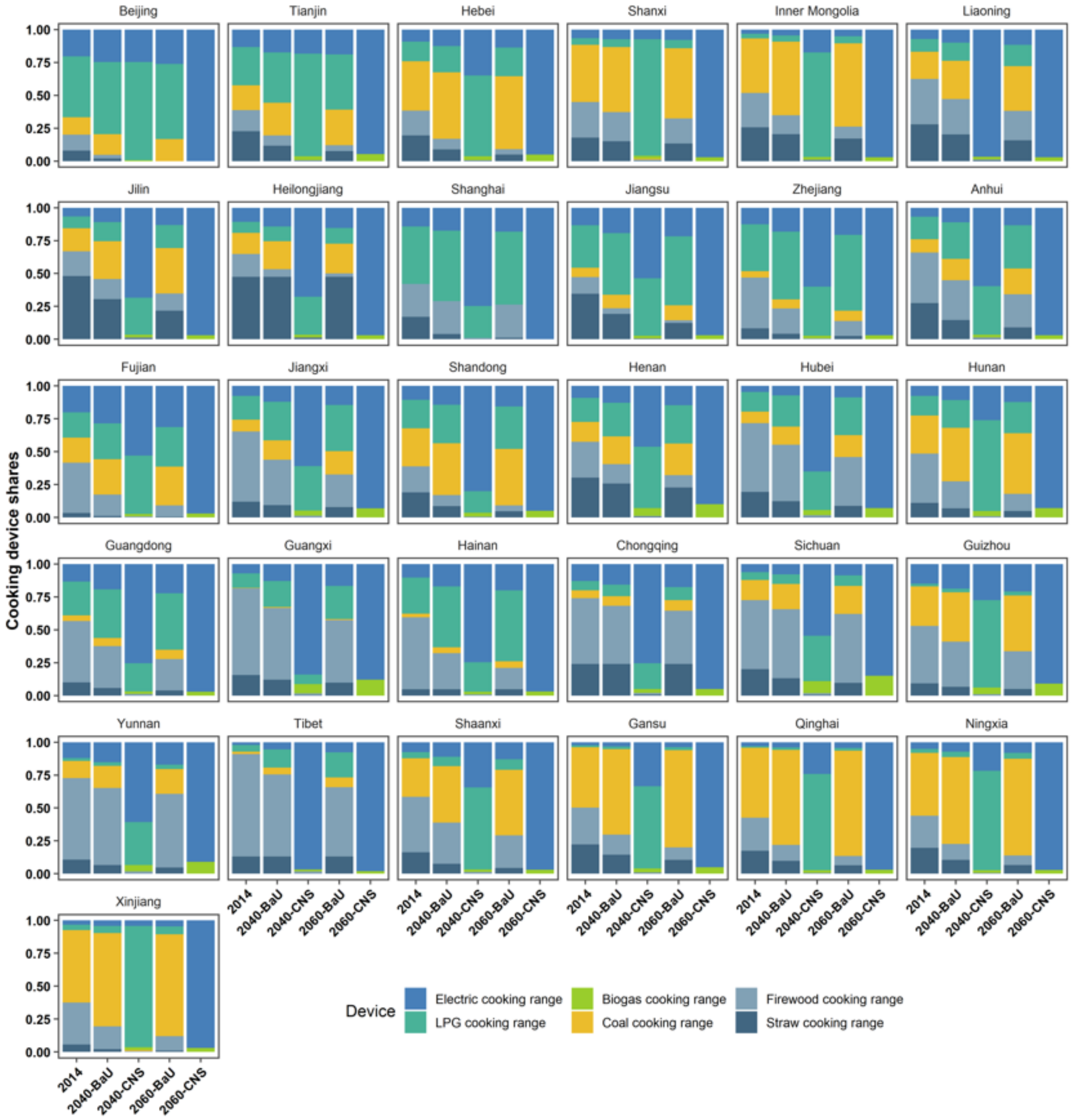

Supplementary Fig 2. Detailed technology shares for rural cooking at the provincial level

Supplementary Fig 3

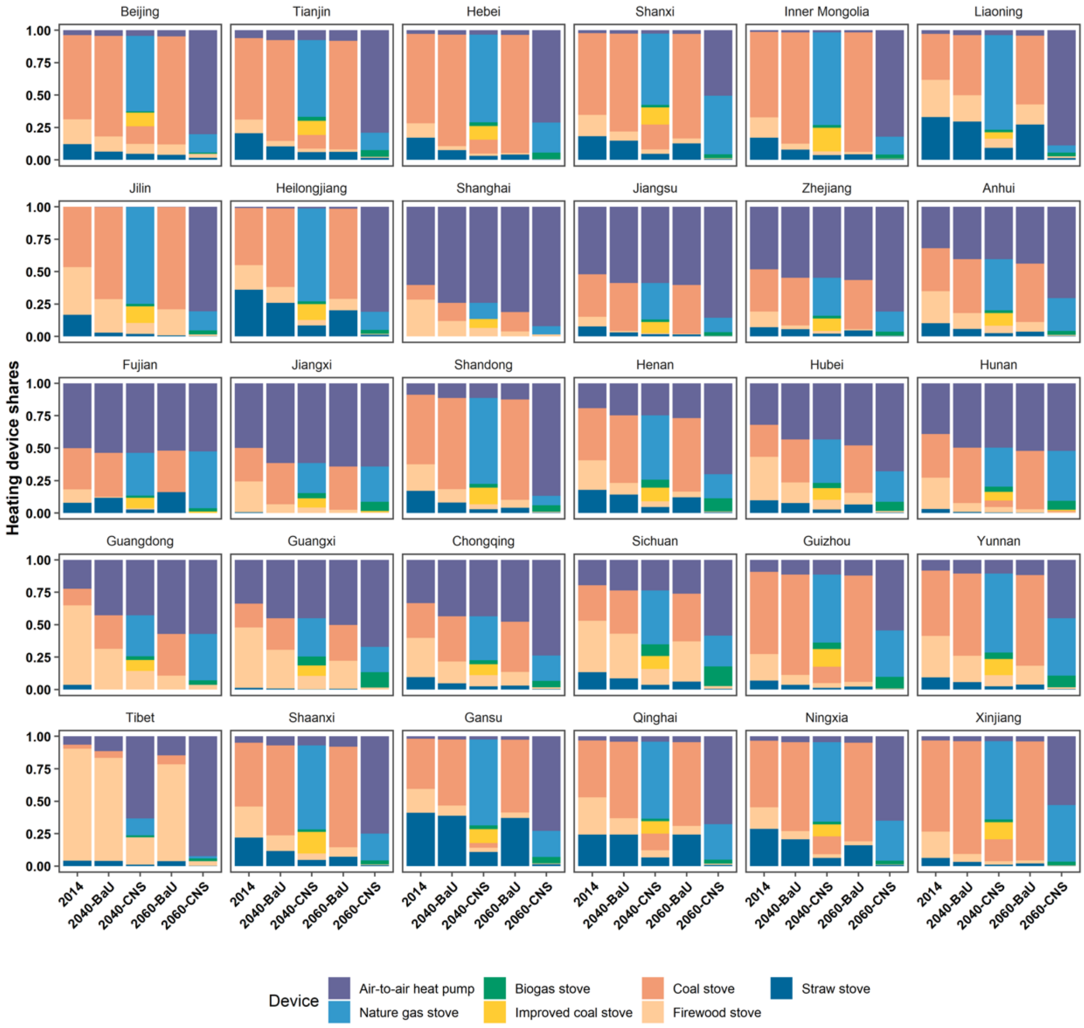

Supplementary Fig 3. Detailed technology shares for rural cooking at the provincial level

Supplementary Fig 4

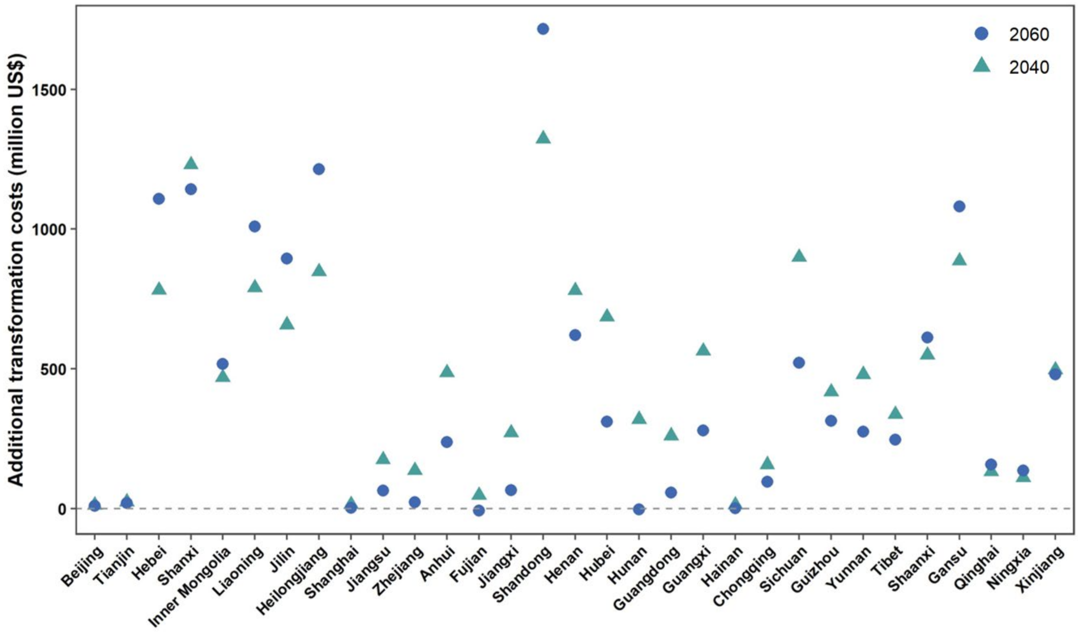

Supplementary Fig 4. Additional transformation costs for each province in 2040 and 2060 (million US$, 2020 price level)

Part C. Sensitivity analysis

C.1 Methods

C.1.1 One-at-a-time and two-at-a-time methods. We applied one-at-a-time and two-at-a-time methods to investigate the alternative impacts of rural residential energy use, system costs, emissions, attributable $PM_{2.5}$ concentrations and related health impacts to changes in key input variables compared with core results. The one-at-a-time method is widely used to explore the sensitivity of results to the change of one input variable while maintaining all other inputs constant[26, 27, 28], where the sensitivity is measured by elasticity ε (Eq. 11). Correspondingly, the two-at-a-time method is used to explore the sensitivity of results to the changes of several input variables while maintaining the other inputs constant, which enables to investigate the effects of different combinations of alternative assumptions on the rural residential energy system and socioeconomic development.

$$\varepsilon = \frac{Output_S - Output_C}{Output_C} \Big/ \frac{Input_S - Input_C}{Input_C} \quad (11)$$

where, $Output_C$ and $Output_S$ are the model outputs of selected indicators under the core scenario and corresponding sensitivity scenario, respectively, while $Input_C$ and $Input_S$ are the model inputs of selected indicators under the core scenario and corresponding sensitivity scenario, respectively.

C.1.2 Key input variables. We conducted a systematic sensitivity analysis with different assumptions on rural socioeconomic, cooking and heating energy and technology systems, health impact parameters to assess the robustness of the related results. The sensitivity analysis was performed covering six groups of key parameters, including (1) different shared socioeconomic pathways, covering people, urbanization and GDP, related to different rural cooking and heating demand at the provincial level; (2) higher or lower contemporary technology capital cost; (3) higher or lower contemporary technology using efficiency; (4) higher or lower contemporary energy price; (5) different exposure-response functions; (6) higher or lower VSL. These parameters affect rural residential energy use, system costs, emissions, attributable $PM_{2.5}$ concentrations and related health impacts. The full suite of input parameters used in the sensitivity analysis is described below.

① **Rural socioeconomic development**. Corresponding to SSP2 pathway assumptions in BaU and CNS, we used two alternative assumptions from the well-established SSP framework[10, 29, 30, 31], the sustainability pathway' (SSP1)[32] and the 'regional rivalry pathway' (SSP3)[33], to cover the range of uncertainty in future rural people and GDP per capita. Rural people size and GDP per capita are key factors in estimating rural cooking and heating demand. Therefore, SSP1 assumes a less rural people[10] and faster GDP growth[11] relating to lower rural cooking and heating

demand. On the contrary, SSP3 indicates a more rural people[10] and lower GDP growth[11] relating to higher rural cooking and heating demand scenario. In order to estimate the elasticity of outputs to rural socioeconomic development, we set the rural people as the input indicator in Eq. 11.

② **Contemporary technology capital cost**. In our study, the model setting of the contemporary technology cost decreased by 20% from 2020 to 2060. While, for the sensitivity analysis, we set the contemporary technology capital cost with the ±20% changes compared with the core scenarios from 2020-2060[13].

③ **Contemporary technology efficiency**. Two alternative contemporary technology efficiency levels are set, including 10% lower (Low technology efficiency)[8] and 10% higher (High technology efficiency) than that under the core scenarios.

④ **Contemporary energy price**. The future expectation for contemporary energy price trajectory is based on previous studies for the core scenarios[14, 15]. The price setting for sensitivity analysis of the two types of contemporary energy (NG/LPG and electricity) was assumed with 20% lower (Low energy price) and 20% higher (High energy price) than that under the core scenario.

⑤ **Exposure-response function**. Previous studies have shown that choosing exposure-response functions (ERFs) may lead to uncertainties in assessing $PM_{2.5}$-related health impacts. In the core scenarios, the Global Exposure Mortality Model (GEMM)[34] is used in our study, and the health outcomes of the log-linear form function[35] and the integrated exposure-response (IER) function[36] are used for the sensitivity analysis of health impact, respectively.

⑥ **The value of statistical life.** The value of statistical life is the product of an individual's willingness to pay to avoid the risk of death from certain people and the inverse of the people's risk reduction. The core scenarios use the medium VSL and the sensitivity analysis use the high and low level of VSL[21].

⑦ **Combination**. We set 64 combinations of the above six group parameters following the two-at-a-time method (Supplementary Data 2). In particular, we emphasize the promotion of electricity or NG/LPG when combining different types of variables in residential energy systems. For instance, in the CNS_SSP1_EFoE&G[H]_ICoE&G[L]_EPoE&G[L] scenario, the socioeconomic settings in CNS under SSP2 switch to SSP1(resulting in lower rural cooking and heating demand), high efficiency of technologies using electricity and NG/LPG (EFoE&G[H]), low initial capital cost of technologies using electricity and NG/LPG (ICoE&G[L]), low energy price of electricity and NG/LPG (EPoE&G[L]). The same scenario naming rules apply to one-at-a-time and two-at-a-time sensitivity scenarios, as shown in Supplementary Data 1 and 2.

C.1.3 Sensitivity scenarios. 52 and 64 sensitivity scenarios based on the one-at-a-time and two-at-a-time methods are shown in Supplementary Table 10 and Supplementary Data 1 and 2.

Supplementary Table 10. Assumptions for sensitivity analysis using the one-at-a-time method

			BaU	CNS
Rural socioeconomic development	Cooking and heating service demand	SSP1	√	√
		SSP3	√	√
Contemporary technology efficiency	Electric cooking range	High	√	√
		Low	√	√
	LPG cooking range	High	√	√
		Low	√	√
	Air-to-air heat pump	High	√	√
		Low	√	√
	Natural gas stove	High	√	√
		Low	√	√
Contemporary technology cost	Electric cooking range	High	√	√
		Low	√	√
	LPG cooking range	High	√	√
		Low	√	√
	Air-to-air heat pump	High	√	√
		Low	√	√
	Natural gas stove	High	√	√
		Low	√	√
Contemporary energy price	Electricity	High	√	√
		Low	√	√
	NG/LPG	High	√	√
		Low	√	√
Health impacts	Exposure-response function	linear	√	√
		non-linear IER	√	√
	Value of statistical life	High	√	√
		Low	√	√

Note: 52 additional sensitivity scenarios are generated based on the one-at-a-time method.

C.2 Results

We analyzed China's rural cooking and heating energy use, CO_2 and SO_2 emissions, and energy system costs in 2060 under 108 sensitivity scenarios by applying the residential module of IMED|TEC. The detailed results are shown in Supplementary Fig 5, Supplementary Data 3 and 4, respectively. Notably, all the above indications are relatively sensitive to rural socioeconomic development. In addition, total energy use, electricity use and SO_2 emissions are moderately sensitive to the efficiency of the electric cooking range, while energy system cost is relatively sensitive to the capital cost of AAHP (Supplementary Data 3 and 4). Nonetheless, the model results still maintain marked differences between the corresponding sensitivity scenarios in BaU and CNS,

i.e., China's RCH consistently would use more contemporary energy and emits less CO_2 and air pollutants while paying higher system costs for achieving carbon neutrality in 2060 (Supplementary Fig 5). Sensitivity analysis results under the combinations of rural socioeconomic development, technology efficiency, contemporary technology capital cost and contemporary energy price would lead to more sensitive results compared with other scenarios. Therefore, we investigated the $PM_{2.5}$ reductions in two extreme carbon neutral scenarios by applying GAINS and found the provincial $PM_{2.5}$ reductions differ little compared with the core CNS scenario. Furthermore, we analyzed the avoided $PM_{2.5}$-associated premature deaths under two additional exposure-response functions and corresponding monetized benefits based on higher or lower VSL.

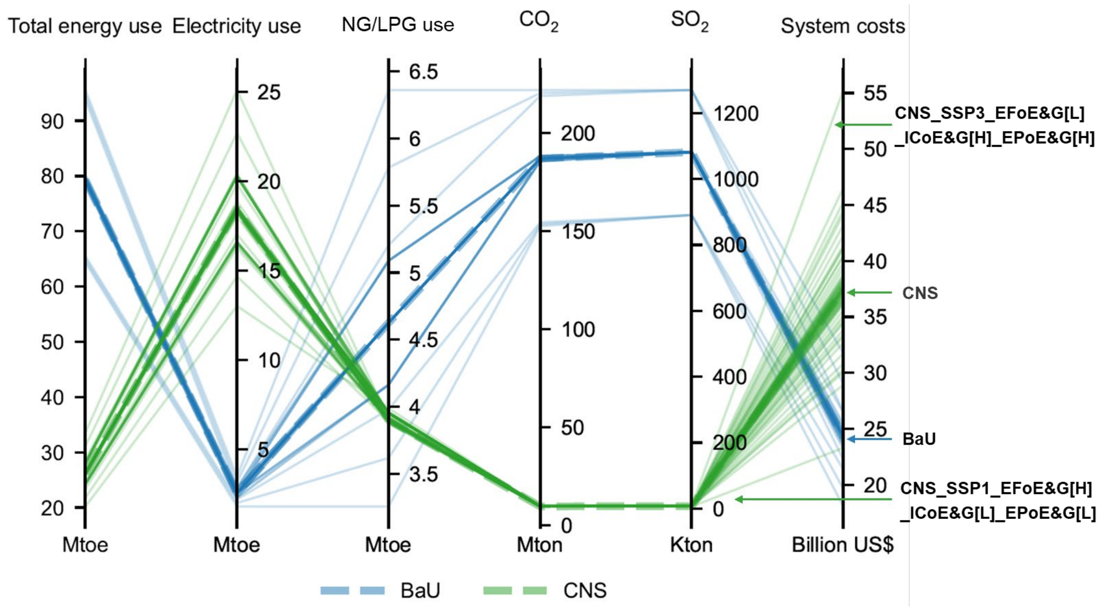

Supplementary Fig 5. Sensitivity analysis results in China's rural residential sector under different alternative scenarios in 2060. Note: The six indicators represent total energy use, electricity use, NG/LPG use, CO_2, SO_2 emissions and energy system cost of China's rural cooking and heating, respectively. There are multiple lines for each core scenario due to different assumptions for the uncertain parameters, including rural socioeconomic development, the efficiency and capital cost of contemporary technologies, and their various combinations (Supplementary Data 2).

C.2.1 Sensitivity analysis of energy use in rural cooking and heating.

All sensitivity scenarios results show that rural socioeconomic development is the most sensitive parameter affecting China's total energy, electricity, and NG/LPG use in 2060. For the sensitivity analysis of the rural people changing from SSP2 to SSP1 in BaU, the elasticities for the total energy use, electricity use and NG/LPG use are 0.855, 1.007, and 1.028, respectively and in CNS, they are 0.79, 0.965, and 0.01, respectively. In contrast, in BaU, the related elasticities are 0.768, 0.951 and 0.993, respectively, when switching rural people from SSP2 to SSP3. In CNS, they are 0.752, 0.916 and 0.004, respectively. Notably, CO_2 emissions are mainly from NG/LPG combustion, so the carbon emission cap limits the maximum use of these energies. Therefore, the lower price of

NG/LPG and associated technologies have negligible effects on the rural energy systems in 2060.

Among all parameters in rural residential energy systems, the efficiency of the electric cooking range is the most sensitive parameter to total energy use and electricity use in CNS, where a 10% increase or decrease would result in a 5.4% (1.41 Mtoe) decrease or 5.46% (1.43 Mtoe) increase in total energy use, 7.68% (1.42 Mtoe) decrease or 7.5% (1.38 Mtoe) increase in electricity use, respectively. The second sensitive parameter is the efficiency of AAHP, but the elasticity is only around 1/3 of the former. However, NG/LPG use is less sensitive to all parameters in rural residential energy systems and elasticities of all parameters below 0.04. Notably, the changes in contemporary technology cost and contemporary energy prices have little impact on contemporary energy use. Therefore, increasing the electric cooking range is most beneficial for reducing RCH energy use.

The combinations of multiple parameters may lead to more sensitive results. Especially in the most sustainable combination (CNS_SSP3_EFoE&G[L]_ICoE&G[H]_EPoE&G[H], when switching people and economic development pathway from SSP2 to SSP1, increasing the efficiency and decreasing the capital cost of electric cooking range and AAHP, decreasing contemporary energy price), the results illustrate that the total energy use, electricity use, and NG/LPG use will decrease by 22.81% (5.97 Mtoe), 29.42% (5.43 Mtoe) and increase by 2.26% (0.09 Mtoe), respectively in 2060 compared with the core scenario. Conversely, in the most unsustainable combination (CNS_SSP1_EFoE&G[H]_ICoE&G[L]_EPoE&G[L]) under the CNS scenario, the total energy use, electricity use, and NG/LPG use will increase by 28.16% (7.37 Mtoe), 36.06% (6.65 Mtoe), and 0.11% (0.004 Mtoe), respectively, compared with the core scenario in 2060 (Supplementary Fig 5). Moreover, we further investigate the alternative air quality impacts under the above two extreme sensitivity scenarios by applying GAINS model (The detailed information refers to "4. Sensitivity analysis on air quality, health impacts and monetized benefits").

C.2.2 Sensitivity analysis of CO_2 and air pollutant emissions.

In BaU, the results of corresponding sensitivity scenarios show that rural socioeconomic development remains the most sensitive parameter affecting China's CO_2 and SO_2 emissions in 2060. Notably, the elasticities of rural socioeconomic development for CO_2 and SO_2 emissions are 0.845 and 0.832 when switching rural people from SSP2 to SSP1. In comparison, the elasticities are 0.709 and 0.691, respectively, for CO_2 and SO_2 emissions when changing rural people from SSP2 to SSP3.

However, in CNS, the corresponding sensitivity scenarios show that the unit changes of almost all parameters have minor impacts on CO_2 emissions (with elasticities less than 0.012). It implies that China's CO_2 emissions in 2060 close to the carbon cap are feasible. Notably, the corresponding sensitivity scenarios in CNS indicate that rural socioeconomic development is the most sensitive parameter affecting SO_2 emissions, with elasticities of 0.541 and 0.553 when the rural people switching to SSP1 and SSP3, respectively. Among all energy system-related parameters, the

efficiency of the electric cooking range is the most sensitive parameter affecting SO_2 emissions, with an elasticity of 0.257.

When combining different groups of parameters, China's CO_2 and SO_2 emissions in 2060 will range from -18.42% to 18.7%, -17.65% to 17.42% in the BaU corresponding sensitivity scenarios, respectively. The corresponding sensitivity scenarios in the CNS have small impacts on China's CO_2 emissions (-0.45%-1.15%). However, China's SO_2 emissions will decrease by 15.59% (1.14 Kton) or increase by 14.13% (1.03 Kton) under the most sustainable and unsustainable combination with changing of rural socioeconomic development, technology efficiency, contemporary technologies capital cost and contemporary energy price.

C.2.3 Sensitivity analysis of energy system costs.

Rural socioeconomic development of corresponding sensitivity scenarios remains the most sensitive parameter affecting RCH energy system cost in 2060 in both BaU and CNS. For instance, in CNS, with rural socioeconomic development switching to SSP1 and SSP3, the elasticity is 0.907 and 0.749, respectively. Among all energy system-related parameters, the capital cost of AAHP is the most sensitive parameter (elasticity of 0.487) in CNS, followed by the price of electricity (elasticity of 0.25).

When combining energy system parameters, the energy system cost will vary from -22.81% to 24.11% (-8.55 to 9.04 billion US$) in CNS compared with core scenarios. The combination parameters include the technology efficiency, capital cost of electric cooking range and AAHP, and contemporary energy price. However, RCH energy system cost will decrease by 37.84% (14.19 billion US$) or increase by 47.42% (17.78 billion US$) under the most sustainable and unsustainable combinations incorporating the rural socioeconomic development.

C.2.4 Sensitivity analysis on air quality, health impacts and monetized benefits.

In the two sensitivity scenarios of CNS_SSP1_EFoE&G[H]_ICoE&G[L]_EPoE&G[L] and CNS_SSP3_EFoE&G[L]_ICoE&G[H]_EPoE&G[H], energy use and air pollutant emissions of China's rural cooking and heating show the most extreme results. Therefore, we input the provincial RCH energy use under the above two scenarios into the GAINS model for investigating the $PM_{2.5}$ reductions compared with BaU. In summary, the provincial $PM_{2.5}$ reductions in the two extreme sensitivity scenarios differ slightly from the core scenarios. Speically, the $PM_{2.5}$ concentration reductions in CNS_SSP1_EFoE&G[H]_ICoE&G[L]_EPoE&G[L] range from -0.27 to 0.13 $\mu g/m^3$ in 2035 and 0 to 0.08 $\mu g/m^3$ in 2050 among all provinces. Correspondingly, the $PM_{2.5}$ concentration reductions in CNS_SSP3_EFoE&G[L]_ICoE&G[H]_EPoE&G[H] range from -0.37 to 0 $\mu g/m^3$ in 2035 and -0.03 to 0.07 $\mu g/m^3$ in 2050 among all provinces.

Due to using different exposure-response functions (ERFs), the avoiding premature deaths vary range from 4.6% to 9.5% in 2035 and from 7.0% to 16% in 2050 (Supplementary Fig 6). The non-

linear integrated exposure-response (IER) function estimates the lowest premature deaths, followed by the Global Exposure Mortality Model (GEMM) function, while the log-linear (LL) function estimates the highest premature deaths. According to the IER function, the CNS scenario will reduce 50.8 thousand (4.8%) and 66.7 thousand (9.1%) premature deaths in 2035 and 2050, respectively. Based on the log-linear function, premature deaths change with a reduction of 108 thousand (9.5%) and 96.9 thousand (16%) in the CNS scenario. It's worth noting that even if switching to IER or log-linear functions, the uncertainty in the health impact assessment may affect the results of the cost-benefit ratio but does not have a decisive impact on the qualitative results.

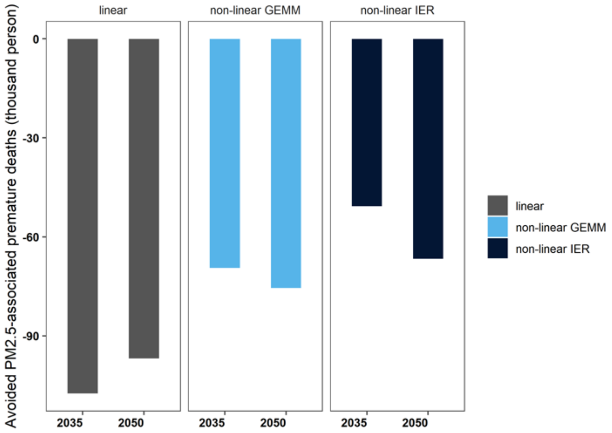

Supplementary Fig 6. Avoided $PM_{2.5}$-associated premature deaths in 2035 and 2050 based on different exposure-response functions

Furthermore, we estimate the monetized health benefits related to avoided $PM_{2.5}$-associated premature deaths based on the latest GEMM at the medium, high and low levels of VSL for each province (Supplementary Table 6). When monetizing health benefits based on the high (low) VSL, China gains national health benefits of 173 (76) and 255(112) billion US$ in 2035 and 2050, respectively. In particular, the benefit-cost ratio of China's RCH decarbonization in 2050 would range from 8.7 to 19.8. At the provincial level, Shandong, Henan and Hebei will gain the most net benefits, whereas Tibet will gain negative net benefits due to extremely low background $PM_{2.5}$ concentrations. However, there have three and two provinces where the health benefits cannot offset the transformation costs in 2035 and 2050 (Supplementary Fig 7).

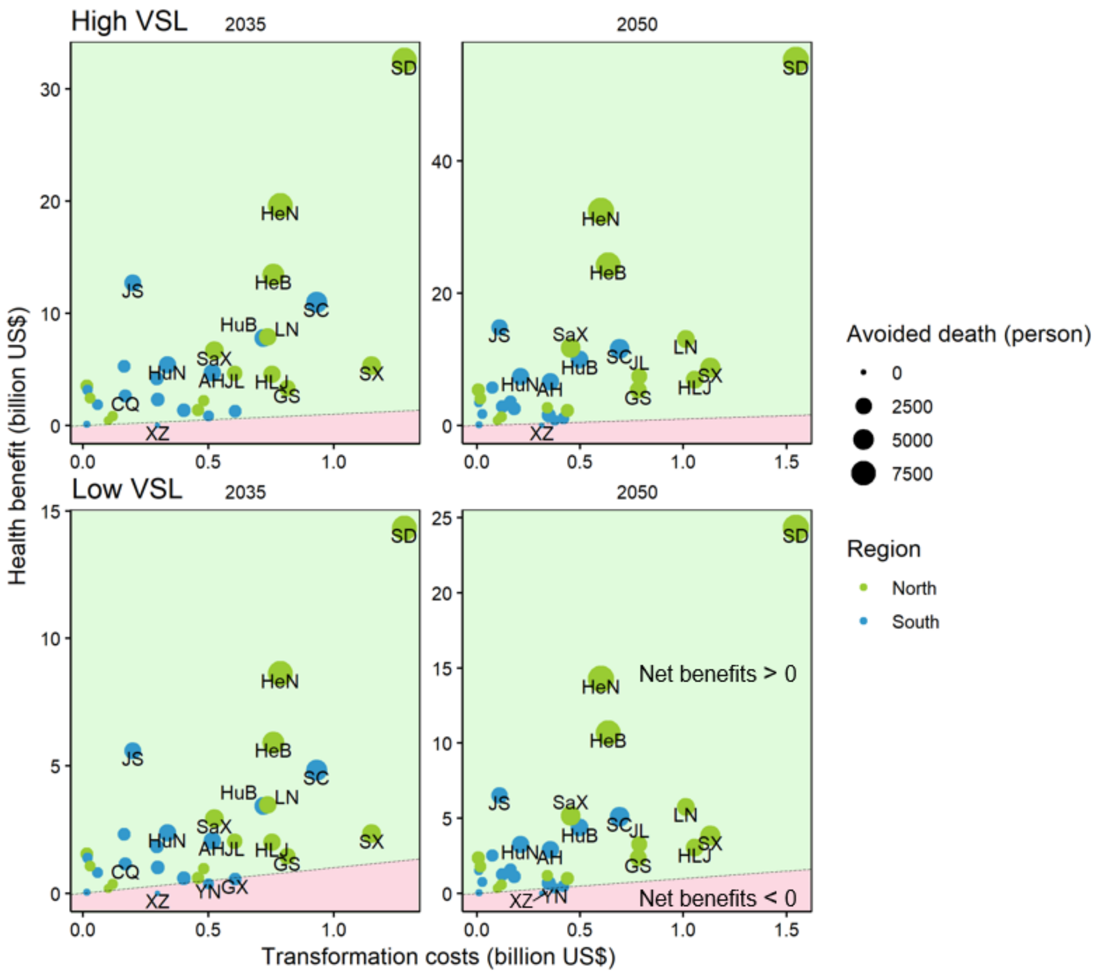

Supplementary Fig 7. Rural cooking and heating transformation costs and monetized health benefits related to avoided $PM_{2.5}$-associated premature deaths at the high and low value of statistical life (VSL) level in 2035 and 2050. Blue dots represent southern provincial regions, while dark green dots represent northern provincial regions. Provinces in the light green background area show positive net benefits (monetized health benefits minus transformation costs), whereas provinces in the pink background area exhibit negative net benefits.

Supplementary Reference

1. Tao S, *et al.* Quantifying the rural residential energy transition in China from 1992 to 2012 through a representative national survey. *Nature Energy* **3**, 567-573 (2018).
2. Zhu X, *et al.* Stacked Use and Transition Trends of Rural Household Energy in Mainland China. *Environmental Science & Technology* **53**, 521-529 (2019).
3. Zhou M, *et al.* Environmental benefits and household costs of clean heating options in northern China. *Nature Sustainability* **5**, 329-338 (2021).
4. Niu S, *et al.* Measurement of effective energy consumption in China's rural household sector and policy implication. *Energy Policy* **128**, 553-564 (2019).
5. Zheng X. *China Household Energy Consumption Research Report.* Science Press (2015).
6. Xing R, Hanaoka T, Masui T. Deep decarbonization pathways in the building sector: China's NDC and the Paris agreement. *Environmental Research Letters* **16**, (2021).
7. Jeuland MA, Pattanayak SK. Benefits and Costs of Improved Cookstoves: Assessing the Implications of Variability in Health, Forest and Climate Impacts. *Plos One* **7**, (2012).
8. Khavari B, Ramirez C, Jeuland M, Nerini FF. A geospatial approach to understanding clean cooking challenges in sub-Saharan Africa. *Nature Sustainability* **6**, 447-+ (2023).
9. Yang X. Current situations and technical routes of rural clean heating (in Chinese). *The 14th session of Building Energy Efficiency Academic Week in Tsinghua University: Clean Heating Forum, Beijing*, (2018).
10. Chen Y, Guo F, Wang J, Cai W, Wang C, Wang K. Provincial and gridded population projection for China under shared socioeconomic pathways from 2010 to 2100. *Scientific Data* **7**, 83 (2020).
11. Jiang T, *et al.* Projection of national and provincial economy under the shared socioeconomic pathways in China. *Progressus Inquisitiones de Mutatione Climatis* **14**, 50-58 (2018).
12. Xing R, Hanaoka T, Kanamori Y, Dai H, Masui T. Energy Service Demand Projections and CO2 Reduction Potentials in Rural Households in 31 Chinese Provinces. *Sustainability* **7**, 15833-15846 (2015).
13. International Energy Agency (IEA). Energy Technology Perspectives 2020. Paris: IEA; 2020. https://www.iea.org/reports/energy-technology-perspectives-2020
14. Cao J, *et al.* The general equilibrium impacts of carbon tax policy in China: A multi-model comparison. *Energy Economics* **99**, (2021).
15. Liu XY, *et al.* Achieving carbon neutrality enables China to attain its industrial water-use target. *One Earth* **5**, 188-200 (2022).
16. Meng W, *et al.* Energy and air pollution benefits of household fuel policies in northern China. *Proceedings of the National Academy of Sciences* **116**, 16773-16780 (2019).
17. Wang C, *et al.* Study on air pollutant emission characteristics and emission reduction potential of civil square briquette. *Journal of Environmental Engineering* **15**, 3253-3261 (2021).
18. Amann M, *et al.* y Reducing global air pollution: the scope for further policy interventions. *Philosophical Transactions of the Royal Society a-Mathematical Physical and Engineering Sciences* **378**, (2020).
19. Burnett R, *et al.* Global estimates of mortality associated with long-term exposure to outdoor fine particulate matter. *Proceedings of the National Academy of Sciences* **115**, 9592-9597 (2018).
20. Lelieveld J, Evans JS, Fnais M, Giannadaki D, Pozzer A. The contribution of outdoor air

pollution sources to premature mortality on a global scale. *Nature* **525**, 367-+ (2015).
21. Jin Y, Zhang S. An Economic Evaluation of the Health Effects of Reducing Fine Particulate Pollution in Chinese Cities. *Asian Development Review* **35**, 58-84 (2018).
22. OECD. *Mortality Risk Valuation in Environment, Health and Transport Policies* (2012).
23. Yun X*, et al.* Residential solid fuel emissions contribute significantly to air pollution and associated health impacts in China. *Science Advances* **6**, (2020).
24. Wu S, Zheng X, Wei C. Measurement of inequality using household energy consumption data in rural China. *Nature Energy* **2**, 795-803 (2017).
25. EFC. Synthesis Report 2020 on China's Carbon Neutrality: China's New Growth Pathway: from the 14th Five Year Plan to Carbon Neutrality.). Energy Foundation China, Beijing, China. (2020).
26. Bajzelj B*, et al.* Importance of food-demand management for climate mitigation. *Nature Climate Change* **4**, 924-929 (2014).
27. Hu YC*, et al.* Food production in China requires intensified measures to be consistent with national and provincial environmental boundaries. *Nature Food* **1**, 572-582 (2020).
28. Hawkins JW*, et al.* High-yield dairy cattle breeds improve farmer incomes, curtail greenhouse gas emissions and reduce dairy import dependency in Tanzania. *Nature Food* **3**, 957-+ (2022).
29. van Vuuren DP*, et al.* The Shared Socio-economic Pathways: Trajectories for human development and global environmental change. *Global Environmental Change-Human and Policy Dimensions* **42**, 148-152 (2017).
30. Samir KC, Lutz W. The human core of the shared socioeconomic pathways: Population scenarios by age, sex and level of education for all countries to 2100. *Global Environmental Change-Human and Policy Dimensions* **42**, 181-192 (2017).
31. Jiang L, O'Neill BC. Global urbanization projections for the Shared Socioeconomic Pathways. *Global Environmental Change-Human and Policy Dimensions* **42**, 193-199 (2017).
32. van Vuuren DP*, et al.* Energy, land-use and greenhouse gas emissions trajectories under a green growth paradigm. *Global Environmental Change-Human and Policy Dimensions* **42**, 237-250 (2017).
33. Fujimori S*, et al.* SSP3: AIM implementation of Shared Socioeconomic Pathways. *Global Environmental Change-Human and Policy Dimensions* **42**, 268-283 (2017).
34. Burnett R*, et al.* Global estimates of mortality associated with long-term exposure to outdoor fine particulate matter. *Proceedings of the National Academy of Sciences* **115**, 9592-9597 (2018).
35. Hoek G*, et al.* Long-term air pollution exposure and cardio- respiratory mortality: a review. *Environmental Health* **12**, 43 (2013).
36. Burnett R, T.*, et al.* An Integrated Risk Function for Estimating the Global Burden of Disease Attributable to Ambient Fine Particulate Matter Exposure. *Environmental Health Perspectives* **122**, 397-403 (2014).
