# Supplementary material for: Costs and health benefits of the rural energy transition to carbon neutrality in China
Source: Nat Commun. 2023 Sep 29;14:6101. doi: 10.1038/s41467-023-41707-7 (PMC10541415; doi:10.1038/s41467-023-41707-7)
Supplement: Supplementary file 3 — Description of Additional Supplementary Files [file 41467_2023_41707_MOESM3_ESM.pdf]

Description of Additional Supplementary Files

File Name: Supplementary Data 1
Description: This sheet provides a comprehensive description of 52 sensitivity scenarios, which are based on the one-at-a-time method. Each additional sensitivity scenario is derived from either the baseline scenario (BaU) or the carbon neutrality scenario (CNS). The one-at-a-time method is a well-established approach that allows for exploring the sensitivity of results to the change in an individual input variable, while keeping all other inputs constant. For example, table cell D2 (BaU_SSP1) represents a specific sensitivity scenario where the rural socioeconomic settings in BaU under SSP2 switch to SSP1(resulting in lower rural cooking and heating demand), while keeping all other parameters unchanged.

File Name: Supplementary Data 2
Description: This sheet provides a comprehensive description of 64 sensitivity scenarios, which are based on the two-at-a-time method. Each additional sensitivity scenario is derived from either the baseline scenario (BaU) or the carbon neutrality scenario (CNS). The two-at-a-time method is used to explore the sensitivity of results to the changes of several input variables while maintaining the other inputs constant, which enables to investigate the effects of different combinations of alternative assumptions on the rural residential energy system and socioeconomic settings. For instance, table cell B64 (CNS_SSP1_EFoE&G[H]_ICoE&G[L]_EPoE&G[L]) represents a specific sensitivity scenario where the socioeconomic settings in CNS under SSP2 transition to SSP1 (resulting in lower rural cooking and heating demand), high efficiency of technologies using electricity and NG/LPG (EFoE&G[H]), low initial capital cost of technologies using electricity and NG/LPG (ICoE&G[L]), and low energy price of electricity and NG/LPG (EPoE&G[L]), while keeping other parameters unchanged. The same scenario naming conventions apply to all two-at-a-time sensitivity scenarios.

File Name: Supplementary Data 3
Description: This sheet presents the results of 52 sensitivity scenarios based on the one-at-a-time method. The results include total energy use, electricity use, NG/LPG (natural gas/liquefied petroleum gas) use, CO_2 emissions, SO_2 emissions, and annual energy system cost. For instance, the column 5 displays the results in BaU_EFoCE[H].

File Name: Supplementary Data 4
Description: This sheet presents the results of 64 sensitivity scenarios based on the two-at-a-time method. The results include total energy use, electricity use, NG/LPG (natural gas/liquefied petroleum gas) use, CO_2 emissions, SO_2 emissions, and annual energy system cost. For instance, the column 63 displays the results in CNS_SSP1_EFoE&G[H]_ICoE&G[L]_EPoE&G[L].
