# Supplementary material for: Costs and health benefits of the rural energy transition to carbon neutrality in China
Source: Nat Commun. 2023 Sep 29;14:6101. doi: 10.1038/s41467-023-41707-7 (PMC10541415; doi:10.1038/s41467-023-41707-7)
Supplement: Supplementary file 5 — Reporting Summary [file 41467_2023_41707_MOESM5_ESM.pdf]

nature portfolio

Corresponding author(s): Yan Ru Fang and Yang Xie

Last updated by author(s): Aug 11, 2023

Reporting Summary

Nature Portfolio wishes to improve the reproducibility of the work that we publish. This form provides structure for consistency and transparency in reporting. For further information on Nature Portfolio policies, see our Editorial Policies and the Editorial Policy Checklist.

Statistics

For all statistical analyses, confirm that the following items are present in the figure legend, table legend, main text, or Methods section.

n/a	Confirmed	
☒	☐	The exact sample size (*n*) for each experimental group/condition, given as a discrete number and unit of measurement
☒	☐	A statement on whether measurements were taken from distinct samples or whether the same sample was measured repeatedly
☒	☐	The statistical test(s) used AND whether they are one- or two-sided<br>*Only common tests should be described solely by name; describe more complex techniques in the Methods section.*
☒	☐	A description of all covariates tested
☒	☐	A description of any assumptions or corrections, such as tests of normality and adjustment for multiple comparisons
☒	☐	A full description of the statistical parameters including central tendency (e.g. means) or other basic estimates (e.g. regression coefficient) AND variation (e.g. standard deviation) or associated estimates of uncertainty (e.g. confidence intervals)
☒	☐	For null hypothesis testing, the test statistic (e.g. *F*, *t*, *r*) with confidence intervals, effect sizes, degrees of freedom and *P* value noted<br>*Give P values as exact values whenever suitable.*
☒	☐	For Bayesian analysis, information on the choice of priors and Markov chain Monte Carlo settings
☒	☐	For hierarchical and complex designs, identification of the appropriate level for tests and full reporting of outcomes
☒	☐	Estimates of effect sizes (e.g. Cohen's *d*, Pearson's *r*), indicating how they were calculated

Our web collection on statistics for biologists contains articles on many of the points above.

Software and code

Policy information about availability of computer code

Data collection	No specific software was used for data collection. The analysis relies on harmonized databases used by a multi-province bottom-up energy system optimization model (IMED|TEC residential module), an air quality model (GAINS) and a health impact assessment model (IMED|HEL). The code base of IMED model has been developed at Peking University and is not available in a publicly shareable version. Code will be made available upon request addressed to the contacts and Hancheng Dai (dai.hancheng@pku.edu.cn). GAINS model is developed by International Institute of Applied Systems Analysis, which is available at https://gains.iiasa.ac.at/gains/.
Data analysis	The software used for the analysis in the IMED|TEC and IMED|HEL model is GAMS (GAMS 32.1.0).<br>The results analysis is done by using the open-source software R-4.0.2 and Python-3.9.15.

For manuscripts utilizing custom algorithms or software that are central to the research but not yet described in published literature, software must be made available to editors and reviewers. We strongly encourage code deposition in a community repository (e.g. GitHub). See the Nature Portfolio guidelines for submitting code & software for further information.

Data

Policy information about availability of data

All manuscripts must include a data availability statement. This statement should provide the following information, where applicable:

- Accession codes, unique identifiers, or web links for publicly available datasets
- A description of any restrictions on data availability
- For clinical datasets or third party data, please ensure that the statement adheres to our policy

Data supporting the findings is available within the Manuscript, Supplementary Information and Supplementary Table. Additionally, we use a representative nationwide rural household energy database conducted by Tao et al.

Research involving human participants, their data, or biological material

Policy information about studies with human participants or human data. See also policy information about sex, gender (identity/presentation), and sexual orientation and race, ethnicity and racism.

Reporting on sex and gender	None
Reporting on race, ethnicity, or other socially relevant groupings	None
Population characteristics	None
Recruitment	None
Ethics oversight	None

Note that full information on the approval of the study protocol must also be provided in the manuscript.

Field-specific reporting

Please select the one below that is the best fit for your research. If you are not sure, read the appropriate sections before making your selection.

☐ Life sciences ☐ Behavioural & social sciences ☒ Ecological, evolutionary & environmental sciences

For a reference copy of the document with all sections, see nature.com/documents/nr-reporting-summary-flat.pdf

Ecological, evolutionary & environmental sciences study design

All studies must disclose on these points even when the disclosure is negative.

Study description	Rural energy transition is critical in China's efforts to achieve carbon neutrality and improve air quality. However, the costs and health benefits associated with the transition to carbon neutrality remain unclear. Using an integrated energy-air quality-health modeling framework, we identify a cost-effective transition pathway, considering air quality-related health impacts. We find that contemporary energy consumption for rural cooking and heating would triple from 2014 to 2060 under carbon neutrality target, leading a substantial narrow-down in energy poverty nationwide. By 2060, electric cooking ranges and air-to-air heat pumps should be widely integrated, costing 13 billion US$ additional transformation costs nationally, with ~40% concentrated in Shandong, Heilongjiang, Shanxi and Hebei provinces. Rural residential decarbonization would remarkably improve ambient air quality in northern China, yielding substantial health co-benefits. Notably, monetized health benefits in most provinces are projected to offset the transformation costs, except for certain relatively under-developed southwestern provinces, implying the need for increased financial support for rural residents in these areas.
Research sample	This study is based on modeling simulation, consisting of a multi-province bottom-up energy system optimization model (IMED|TEC) , an air quality model (GAINS) and a health impact assessment model (IMED|HEL). No sampling process was conducted.
Sampling strategy	Our study is a model study and based on modeling simulation, so sampling strategy is not applicable to this study.
Data collection	The study mainly builds on the model outputs available in IMED|TEC, GAINS and IMED|HEL models. The additionally collected data for model calibration, scenarios and sensitivity analysis collected from literature are listed in Manuscript and Supplementary Information including the reference of the data.
Timing and spatial scale	Our study is a model study, which does not involve experiment and experiment recording time. The residential module of IMED|TEC model takes 2014 as the base year and one year as a cycle until 2060. Therefore, the frequency of simulated energy data is 1 year. GAINS model and IMED|HEL model mainly focus on 2035 and 2050. Our study covers 31 provincial administrative regions in China.

Data exclusions	No data were excluded from our analysis.
Reproducibility	Our study is a model study, which does not involve experiment. All the analyses were based on models and well-defined methods, so the results can be reliably reproduced.
Randomization	Based on an integrated assessment framework (consisting of IMED|TEC, GAINS and IMED|HEL), we set up two representative scenarios to illustrate the transition pathways of rural cooking and heating toward carbon neutrality at the provincial level. Therefore, randomization was not relevant for this study.
Blinding	Based on an integrated assessment framework (consisting of IMED|TEC, GAINS and IMED|HEL), we set up two representative scenarios to illustrate the transition pathways of rural cooking and heating toward carbon neutrality at the provincial level. Therefore, blinding was not relevant for this study.

Did the study involve field work? ☐ Yes ☒ No

Reporting for specific materials, systems and methods

We require information from authors about some types of materials, experimental systems and methods used in many studies. Here, indicate whether each material, system or method listed is relevant to your study. If you are not sure if a list item applies to your research, read the appropriate section before selecting a response.

Materials & experimental systems

n/a	Involved in the study
☒	☐ Antibodies
☒	☐ Eukaryotic cell lines
☒	☐ Palaeontology and archaeology
☒	☐ Animals and other organisms
☒	☐ Clinical data
☒	☐ Dual use research of concern
☒	☐ Plants

Methods

n/a	Involved in the study
☒	☐ ChIP-seq
☒	☐ Flow cytometry
☒	☐ MRI-based neuroimaging
